# DHA–Triacylglycerol Accumulation in Tacrolimus-Induced Nephrotoxicity Identified by Lipidomic Profiling

**DOI:** 10.3390/ijms26157549

**Published:** 2025-08-05

**Authors:** Sho Nishida, Tamaki Ishima, Daiki Iwami, Ryozo Nagai, Kenichi Aizawa

**Affiliations:** 1 Department of Translational Research, Clinical Research Center, Jichi Medical University Hospital, Shimotsuke 329-0498, Japan; 2 Division of Renal Surgery and Transplantation, Department of Urology, Jichi Medical University, Shimotsuke 329-0498, Japan; 3 Jichi Medical University, Shimotsuke 329-0498, Japan; 4 Clinical Pharmacology Center, Jichi Medical University Hospital, Shimotsuke 329-0498, Japan

**Keywords:** calcineurin inhibitor, tacrolimus, lipidomics, docosahexaenoic acid, triacylglycerol profiling, renal injury

## Abstract

Tacrolimus (TAC)-induced chronic nephrotoxicity (TAC nephrotoxicity) remains a major contributor to late allograft dysfunction in kidney transplant recipients. Although detailed mechanisms remain incompletely understood, our previous metabolomic studies revealed disruptions in carnitine-related and redox pathways, suggesting impaired mitochondrial β-oxidation of fatty acids. To further characterize metabolic alterations associated with this condition, we conducted an untargeted lipidomic analysis of renal tissues using a murine model of TAC nephrotoxicity. TAC (1 mg/kg/day) or saline was subcutaneously administered to male ICR mice for 28 days, and kidney tissues were harvested for comprehensive lipidomic profiling. Lipidomic analysis was performed with liquid chromatography–tandem mass spectrometry (*p* < 0.05, *n* = 5/group). Triacylglycerols (TGs) were the predominant lipid class identified. TAC-treated mice exhibited reduced levels of unsaturated TG species with low carbon numbers, whereas TGs with higher carbon numbers and various degrees of unsaturation were increased. All detected TGs containing docosahexaenoic acid (DHA) showed an increasing trend in TAC-treated kidneys. Although accumulation of polyunsaturated TGs has been previously observed in chronic kidney disease, the preferential increase in DHA-containing TGs appears to be a unique feature of TAC-induced nephrotoxicity. These results suggest that DHA-enriched TGs may serve as a metabolic signature of TAC nephrotoxicity and offer new insights into its pathophysiology.

## 1. Introduction

Tacrolimus (TAC), a calcineurin inhibitor, is widely used for immunosuppressive therapy in organ transplantation and autoimmune diseases [1,2,3]. Its potent immunosuppressive properties have made it a cornerstone in clinical transplantation protocols. In kidney transplantation, TAC markedly reduces the rate of acute rejection and enhances short-term graft survival [4]. Due to its efficacy, approximately 90% of immunosuppressive regimens worldwide now rely on TAC as a central agent [5]. TAC exerts its effect by bonding to FK506 binding protein [6]. This complex primarily inhibits calcineurin, a calcium/calmodulin-dependent phosphatase involved in T-cell activation and its proliferation. This inhibition prevents transcription of interleukin-2 and other cytokines, thereby suppressing immune responses. This function makes TAC a key immunosuppressive drug in both solid organ transplantation and autoimmune disease management [7].

Despite its widespread use and effectiveness, TAC-induced chronic nephrotoxicity remains a significant clinical concern. Chronic nephrotoxicity refers to progressive and irreversible renal damage caused by prolonged exposure to TAC [7,8]. This condition, often termed TAC nephrotoxicity, reportedly affects up to 70% of renal transplant recipients within ten years of transplantation [9]. Long-term use of TAC, while beneficial in preventing acute rejection, poses a serious threat to graft longevity. Histopathological features of TAC nephrotoxicity include arteriolar hyalinosis and interstitial fibrosis. These changes are thought to result from a combination of vascular endothelial injury and tubular damage, leading to kidney fibrosis and compromised renal function over time [10]. The dual impact on both vascular and tubular compartments underscores the complexity of TAC-induced renal injury.

Despite these characteristic histological findings, detailed pathophysiological mechanisms underlying TAC nephrotoxicity remain poorly understood. Currently, no effective targeted therapies are available to prevent or reverse this condition [7]. Clinicians are therefore faced with the challenge of balancing short-term immunosuppressive benefits of TAC with its long-term nephrotoxic risks. This clinical dilemma highlights an urgent need for deeper mechanistic insights and development of novel therapeutic strategies.

Recent progress in mass spectrometry-based “omics” technologies has enabled comprehensive profiling of small molecules, providing valuable insights into the pathophysiology of chronic kidney disease (CKD) and its primary etiologies [11,12,13,14]. These technologies, including metabolomics and lipidomics, allow identification of molecular signatures associated with disease states and drug-induced toxicities. In previous work, we applied metabolomic profiling to a murine model of TAC nephrotoxicity and identified disturbances in lipid-related pathways. Specifically, we observed carnitine depletion in renal tissues and reduced levels of NAD^+^ in whole blood samples [15,16]. Carnitine plays a crucial role in fatty acid transport supporting β-oxidation and mitochondrial energy metabolism, whereas NAD^+^ is essential for redox reactions and cellular homeostasis. Depletion of these molecules suggests disruption of energy metabolism and oxidative stress regulation in kidneys continuously exposed to TAC.

Based on these findings, we hypothesized that TAC nephrotoxicity is associated with specific lipidomic signatures in kidney tissue. Lipidomics, a subfield of metabolomics, focuses on comprehensive analysis of lipids and their roles in cellular physiology and pathology. In the present study, we performed a comprehensive lipidomic analysis to further characterize metabolic alterations underlying TAC-induced renal injury.

## 2. Results

### 2.1. Summary of Lipidomic Results

**Figure 1:**
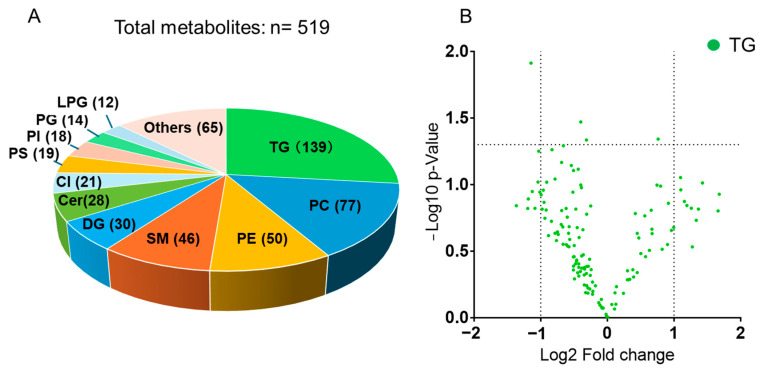
Overview of detected metabolites and distribution of triacylglycerols (TGs). (**A**) We identified 519 lipid metabolites. The number in parentheses following each lipid class indicates the number of detected metabolites in that class. TGs comprised the predominant class (139 species) (*n* = 5/group). (**B**) Volcano plot of TGs showing the log2 fold change (TAC group/control group) on the *x*-axis and the –log10 *p*-value on the *y*-axis. Each green dot represents an individual TG species. The two vertical dotted lines indicate −1 and 1 for Log2 Fold change, respectively. The horizontal dotted line indicates a *p*-value of 0.05. Abbreviations: Cer: ceramide; Cl: cardiolipin; DG: diacylglycerol; LPG: lysophosphatidylglycerol; PC: phosphatidylcholine; PE: phosphatidylethanolamine; PG: phosphatidylglycerol; PS: phosphatidylserine; SM: sphingomyelin; Pl: phosphatidylinositol; TG: triacylglycerol.

A total of 519 lipid metabolites were identified in renal tissues. Among them, triacylglycerols (TGs) were the predominant lipid class, with 139 TG species detected (Figure 1A). Following TGs, lipidomic classes in which more than ten metabolites were detected are as follows: Phosphatidylcholine (PC), 77 species; Phosphatidylethanolamine (PE), 50 species; Sphingomyelin (SM), 46 species; Diacylglycerol (DG), 30 species; Ceramide (Cer), 28 species; Cardiolipin (Cl), 21 species; Phosphatidylserine (PS), 19 species; Phosphatidylinositol (PI), 18 species; Phosphatidylglycerol (PG), 14 species; Lysophosphatidylglycerol (LPG), 12 species. Lipid classes in which fewer than ten metabolites were detected, were classified as “Others.” The “Others” class contained the following lipids: acyl carnitine, bisphosphate, campesterol ester, cholesterol esters, coenzyme Q, fatty free acids (FFAs), globotriosyl ceramide, glucosyl ceramide, lactosyl ceramide, lysophosphatidylethanolamine, lysophosphatidylinositol, monoglyceride, sitosterol ester, sulfatide. Detailed numbers of metabolites detected in each lipid class are presented in Supplemental Table S1. Notably, carnitine was undetectable, and only two acylcarnitine species were identified (Supplemental Table S1). The ratio of average relative area values for TGs in the TAC-treated and control groups is shown in Figure 1B with volcano plot. There was no consistent overall trend toward either increased or decreased TG levels among all TG classes.

### 2.2. Distribution and Trends of Detected TGs

**Figure 2:**
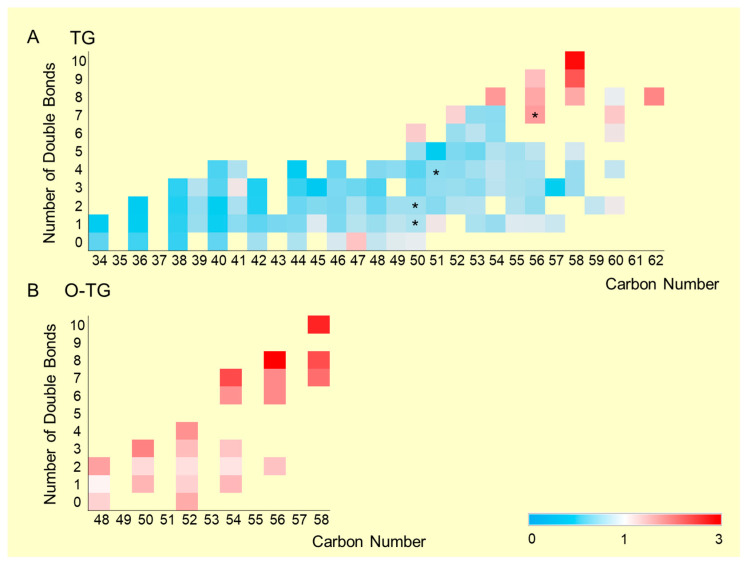
Heatmaps of triacylglycerol (TG) profiles categorized by carbon numbers and numbers of double bonds. (**A**) Relative abundance of TG species based on carbon number and unsaturation. (**B**) Ether-linked TG species analyzed separately due to overlapping classification. The yellow background color in this figure represents the TG blank. Red indicates an increase (TAC/Control ratio > 1), and blue indicates a decrease (TAC/Control ratio < 1). The *x*-axis shows the total carbon number. The *y*-axis shows the number of double bonds. The ratio was calculated as follows: the relative ratio = average relative areas of the TAC group/average relative areas of the control group (* *p* < 0.05). TG, triacylglycerol; O-TG, ether-linked triacylglycerol.

Heatmaps illustrating distribution trends of TG species, categorized by total carbon numbers and numbers of double bonds, are presented in Figure 2A,B. Ratios represent average relative areas in the TAC group relative to the control group. Ether-linked TG species are shown separately in Figure 2B due to overlapping classifications. Numbers of double bonds ranged from 0 to 10 in this study. Total carbon numbers varied from 34 to 62. In general, TGs with lower carbon numbers and fewer double bonds decreased in the TAC group, whereas TGs with higher carbon numbers and greater numbers of double bonds increased.

### 2.3. TGs Containing Major Polyunsaturated Fatty Acids (PUFAs)

**Figure 3:**
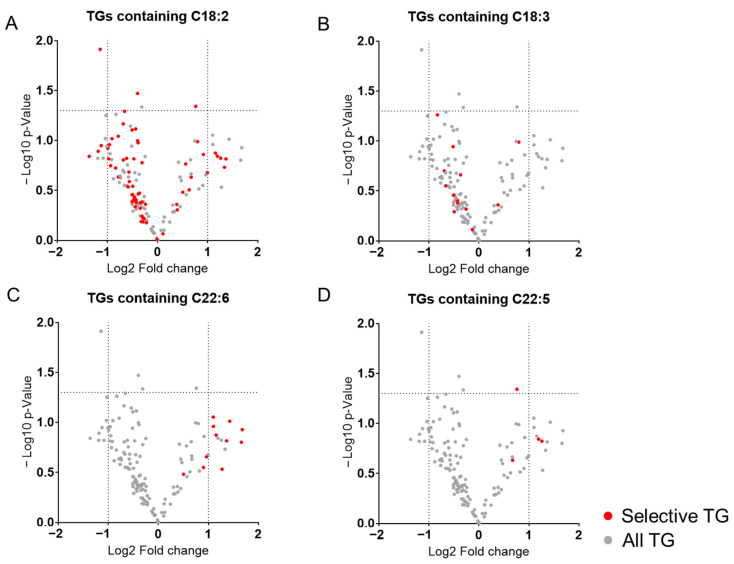
Volcano plots of TGs containing representative PUFAs. (**A**) TGs containing linoleic acid (C18:2); (**B**) linolenic acid (C18:3); (**C**) docosahexaenoic acid (DHA, C22:6); (**D**) docosapentaenoic acid (DPA, C22:5). Gray dots represent all TGs; red dots indicate TGs containing the specified PUFA. The *x*-axis shows the log2 fold change (TAC/Control); the *y*-axis shows the −log10 *p*-value. The two vertical dotted lines indicate −1 and 1 for Log2 Fold change, respectively. The horizontal dotted line indicates a *p*-value of 0.05. TG; triacylglycerol, PUFA; polyunsaturated fatty acid, C18:2; linoleic acid, C18:3; linolenic acid, C22:5; DPA, C22:6; DHA.

Figure 3 shows volcano plots of TGs containing representative polyunsaturated fatty acids (PUFAs). No clear accumulation was observed for TGs containing linoleic acid (C18:2) or linolenic acid (C18:3) (Figure 3A,B). In contrast, all detected TGs containing docosahexaenoic acid (DHA, C22:6) tended to increase in the TAC group (Figure 3C). TGs containing docosapentanoic acid (DPA, C22:5) were also elevated in the TAC group, though only a few species were detected (Figure 3D). These findings suggest a selective accumulation of TGs containing highly PUFAs in TAC-induced nephrotoxicity, particularly those incorporating DHA.

## 3. Discussion

Building upon our previous investigations of nephrotoxic effects of TAC, in this study, we conducted a lipidomic analysis to identify alterations in lipid metabolism in renal tissues subjected to TAC exposure. Lipidomics provides a powerful approach to comprehensively examine lipid species in biological samples, and it is generally categorized into untargeted and targeted analyses [17,18,19]. Although targeted lipidomics enable accurate quantification of known lipid molecules, untargeted lipidomics offer a broader view by profiling a wide spectrum of lipids, including unexpected or novel species. Since our objective was to capture global metabolic disturbances associated with TAC nephrotoxicity, we employed an untargeted approach to assess lipid perturbations in murine kidney tissues.

Our lipidomic profiling revealed that TGs constituted the most abundant lipid class in kidney tissues examined (Figure 1A). TGs serve not only as energy reserves, but also contribute to cellular processes such as maintenance of membrane structure, intracellular signaling, and metabolic regulation [14]. Importantly, TGs are implicated in systemic metabolic disorders such as atherosclerosis and metabolic syndrome—conditions that share clinical and mechanistic similarities with adverse effects of TAC [7]. Thus, characterizing TG behavior under TAC exposure may provide insights into renal metabolic adaptations or pathologies elicited by the drug.

While overall TG levels did not exhibit a consistent trend between the TAC and control groups (Figure 1B), a more detailed examination revealed a shift in TG species composition. Specifically, TGs with lower total carbon numbers and fewer double bonds tended to decrease in the TAC group (Figure 2A). Conversely, TGs with higher carbon numbers and more double bonds exhibited a relative increase.

Lipidomic analysis of the relationship between CKD and TG has been performed on human and animal samples, and several previous reports have shown trends similar to the present results [17,20,21,22,23,24]. Some have indicated that in CKD patients, saturated FFAs and TGs containing PUFAs accumulate in plasma as CKD progresses [17,24]. To date, there have been no reports of lipidomic analysis using renal tissue samples from CKD patients. Although our results were obtained using renal tissue from mice, PUFA-containing TGs were elevated in kidney tissue with TAC nephrotoxicity. Our results suggest that trends in plasma TGs reflect lipidomic TG changes occurring in kidney tissue.

The increase in PUFA-containing TGs in plasma has been described as a compensatory reaction to decreased levels of FFAs in tissues [19,24]. It has been reported that the process of energy compensation through accelerated β-oxidation collapses due to decreased mitochondrial function associated with CKD progression. Afshinnia et al. showed that as CKD progresses, PUFA-containing TGs are upregulated in human plasma [24]. Moreover, several reports show that lipotoxicity of saturated FFAs can be attenuated by accumulating PUFA-containing TGs, which serve as stable, less toxic lipid reservoirs [19,25]. Focusing on TAC nephrotoxicity, a previous report showed that fatty acid metabolism was downregulated in rats [26]. In addition, we previously reported the possibility of impaired β-oxidation in renal tissues due to carnitine deficiency in TAC nephrotoxicity [15]. Abdulwahab et al. also found TAC-induced nephrotoxicity with mitochondrial oxidative stress in mice [27]. These findings suggest that TAC nephrotoxicity may disrupt β-oxidation, causing subsequent accumulation of PUFA-containing TGs in renal tissues.

PUFA-containing TGs are elevated in blood samples from patients with advanced CKD and diabetic kidney disease [23,24], but it is not known which PUFA-containing TG species increase. Our results revealed that not all PUFA-containing TGs were elevated in the TAC group. Although linoleic acid- and linolenic acid-containing TGs were detected in abundance in our analysis, there were no clear increasing trends for either of these (Figure 3A,B). Rather, TGs containing DHA were notably increased (Figure 3C). DHA, a highly unsaturated FFA, is known for its antioxidant effects in tissues [28]. Focusing on CKD, Koh et al. showed that high plasma DHA levels reduce the risk of CKD [29]. Another study also found that DHA enrichment in plasma can protect kidney function [30]. Interestingly, DHA-containing TGs in plasma also positively increase the estimated glomerular filtration rate [31]. In our study, DPA-containing TGs were also detected with higher tendency in the TAC group. Since DPA is an intermediate product of DHA in bioconversion pathways of n-3 PUFA families [32], a similar increase in DPA-containing TGs may be related to that of DHA. It remains unclear whether the increase in DHA-containing TGs in the TAC group reflects a compensatory mechanism for elevated saturated FFAs or impaired metabolic utilization of DHA. The observed increase in TGs containing highly unsaturated PUFAs may be a characteristic feature of TAC-induced nephrotoxicity and merits further investigation.

This study had several limitations. First, the lipidomic analysis used was untargeted, meaning that it did not provide quantitative data on specific lipid species. To confirm and expand upon these findings, future studies should use targeted lipidomics, which can measure individual metabolites more accurately. Additionally, it is still unclear whether the buildup of DHA-containing TGs in kidney tissue is directly related to TAC nephrotoxicity. More research is needed to understand the biological mechanisms behind this lipid accumulation, including identifying enzymes and genetic factors that may be involved in this phenomenon. Another important point is to examine human kidney samples to validate whether the same lipidomic changes occur in humans. Since this study was performed using a mouse model, confirming the present results in human tissues is necessary to understand their clinical relevance.

Despite these limitations, this study is the first application of untargeted lipidomic analysis to kidney tissue in a model of TAC nephrotoxicity. This lipidomic analysis will offer a valuable starting point for future investigations into TAC nephrotoxicity. Future studies should analyze gene and protein expression in kidney tissues using transcriptomic and proteomic approaches, as well as long-term metabolic studies, to better understand how TAC causes chronic kidney injury. These approaches will help clarify the disease process and may lead to new treatments to protect kidney function in kidney transplantation patients who rely on TAC as a key immunosuppressive drug.

## 4. Materials and Methods

### 4.1. Sample Collection

In this study, a mouse model with chronic TAC nephrotoxicity was developed based on methods used in several previous reports [15,33]. The mouse model has been validated in renal fibrosis and tubular injury by quantitative PCR of established fibrosis and tubular injury markers [15]. Sample size was decided based on previous reports using similar animal models [15,16,33,34,35,36,37]. Briefly, ten 7-week-old male ICR mice were housed in mouse cages (Maxi-Miser^®^ Caging System #5, Oriental Giken Corporation, Tokyo, Japan) with a 12/12 h light/dark cycle. Humidity was maintained at 40 ± 10% and 23 ± 2 °C. Mice were furnished with a low-sodium diet (0.01% sodium, CLEA Japan, Inc., Tokyo, Japan). They were separated randomly into two groups after 7 days administration (n = 5 per group). The TAC group received an osmotic pump (ALZET^®^ osmotic pumps 2004, ALZET Osmotic pumps, Cupertino, CA, USA), and TAC (Prograf^®^, Astellas Pharma Inc., Tokyo, Japan) was administered subcutaneously at 1 mg/kg/day referencing previous reports [15,33]. The control group received normal saline using the same protocol. Samples were collected after 28 days of continuous subcutaneous administration. Under isoflurane anesthesia (induction concentration; 4–5%, maintenance concentration; 2–3%), mice were placed in a supine position and a laparotomy was performed via midline abdominal incision. Kidneys were immediately dissected, and 20–30 mg samples were maintained on ice, before being frozen and stored at −80 °C until lipidomic analysis. This animal protocol was approved by the Animal Welfare Committee of Jichi Medical University (protocol code: 23008-01, 3 July 2023).

### 4.2. Lipidomic Analysis

#### 4.2.1. Materials for Lipid Analysis

All standards were purchased from Avanti Polar Lipids (Birmingham, AL, USA). Methanol and isopropanol of LC/MS quality, and chloroform of JIS special grade were obtained from Kanto Chemical Co., Inc. (Tokyo, Japan). Ultrapure water was from a Milli-Q water system (Millipore, Milford, MA, USA).

#### 4.2.2. Lipid Preparation

Lipidomic analysis was performed following the Lipidome lab Non-targeted Lipidome Scan package (Lipidome Lab, Akita, Japan) using liquid chromatograph orbitrap mass spectrometry (LC-OrbitrapMS, Thermo Fisher Scientific, Waltham, MA, USA), based on methods described previously [38,39]. Briefly, kidney samples were weighed, methanol was added to achieve a concentration of 25 mg/mL, and samples were crushed using a bead homogenizer. A suspension was then prepared. Forty microliters of suspension were collected corresponding to 1 mg of tissue weight. The collected suspension was extracted for total lipids using a modified Bligh and Dyer method with chloroform, methanol, and water. The extracted lipid fraction was dried with nitrogen gas. After this treatment, it was redissolved in 1 mL of methanol. For LC-MS/MS analysis, a 10 µL aliquot of this solution was injected, corresponding to approximately 10 µg of tissue.

#### 4.2.3. Lipidomic Analysis by LC-MS/MS

Lipidomic profiling was performed using a high-resolution LC–MS/MS system comprising a Q-Exactive Plus Orbitrap mass spectrometer coupled with an UltiMate 3000 liquid chromatography system (Thermo Fisher Scientific, Waltham, MA, USA). Chromatographic separation was achieved using a metal-free L-column3 C_18_ column (2.0-µm particle size, 2.0 × 100 mm inner diameter, CERI, Tokyo, Japan), maintained at a constant temperature of 40 °C. Buffer A was a mixture of 2-propanol, methanol, and water in a volumetric ratio of 5:1:4, supplemented with 5 mM ammonium formate and 0.05% ammonium hydroxide (28% in Milli-Q water). Buffer B consisted of isopropanol containing the same concentrations of ammonium formate and ammonium hydroxide as mobile phase A.

A gradient elution program was applied to optimize lipid separation. The gradient began with 40% Buffer B at 0 min, increased linearly to 60% B over 1 min, then to 80% B over the next 8 min. This was followed by a ramp to 95% B over 2 min, which was held for 11 min. Subsequently, the gradient was reduced to 5% B over 0.1 min and maintained for 2.9 min. Finally, the gradient was increased back to 40% B over 0.1 min and held for 4.9 min to re-equilibrate the column. The total run time was 30 min. The flow rate was set at 0.1 mL/min, and the injection volume for each sample was 10 µL.

Mass spectrometric detection was carried out using a heated electrospray ionization (HESI-II) source operated in both positive and negative ionization modes. Source parameters were optimized to ensure efficient ionization and stable spray conditions. Sheath gas and auxiliary gas flow rates were set to 60 and 10 arbitrary units, respectively, while sweep gas was not applied (0 arbitrary units). The spray voltage was set to +3.2 kV for positive mode and −3.0 kV for negative mode. The ion transfer capillary temperature was maintained at 300 °C in positive mode and −320 °C in negative mode, while the heater temperature was set at 325 °C for both modes. The S-lens RF level was adjusted to 50 to optimize ion transmission.

The Orbitrap mass analyzer was operated in full-scan mode with a resolving power of 70,000. The scan range was set from 200 to 1800 m/z in positive ion mode and from 190 to 1800 m/z in negative ion mode. The automatic gain control (AGC) target was set to 1 × 10^5^ for positive mode and 3 × 10^6^ for negative mode to ensure optimal ion accumulation. For tandem mass spectrometry (MS/MS), a data-dependent acquisition (DDA) strategy was employed, targeting the 20 most intense precursor ions. MS/MS scans were performed at a resolving power of 17,500 in positive mode and 35,000 in negative mode. Fragmentation was achieved using stepped normalized collision energies of 20, 30, and 40. The isolation window was set to 4.0 m/z, and the AGC target for MS/MS scans was 1 × 10^5^. A dynamic exclusion time of 10.0 s was applied to prevent repeated fragmentation of the same precursor ion.

Raw data files generated from the LC-MS/MS analysis were processed using Lipid Search software, version 5.1.6 (Mitsui Knowledge Industries Co., Ltd., Tokyo, Japan). This software enables identification of intact lipid species based on accurate mass measurements and MS/MS fragmentation patterns, including head-group and fatty acid composition. Due to the complexity of biological matrices and the diversity of lipid species, it is not feasible to normalize matrix effects across all detected peaks. This limitation arises from the inability to prepare internal standards for every lipid species present. Therefore, relative quantification was performed by calculating the ratio of the chromatographic peak area of each lipid analyte to the total peak area of all detected lipid species.

The quantification and annotation methodology employed in this study adheres to guidelines established by the Lipidomics Standards Initiative. Specifically, quantification corresponds to “Absolute Quantification Level 4,” which denotes relative quantification without internal standards. The structural annotation level is classified as “Fatty Acyl/Alkyl Level or Hydroxyl Group Level,” indicating that lipid species were identified based on their fatty acid or alkyl chain composition and hydroxyl groups [40].

### 4.3. Statistical Analysis and Graphic Design

Statistical analysis of metabolites measured by LC-MS/MS was performed with Welch’s *t*-test in R (R: A language and environment for statistical computing. Vienna, Austria. URL: https://www.R-project.org/, accessed on 5 February 2025). *p* < 0.05 was considered statistically significant. The ratio of each lipid metabolite in TAC and control groups was calculated as follows: ratio = average relative area of the TAC group/average relative area of the control group. Volcano plots were generated with GraphPad Prism (ver. 7.00, GraphPad Software, Boston, MA, USA). Heat maps were created using Microsoft Excel (Microsoft Corp., Redmond, WA, USA) based on ratios calculated from analyzed data.

## 5. Conclusions

Untargeted lipidomic analysis of renal tissues in a murine model of tacrolimus-induced nephrotoxicity revealed an altered TG profile, characterized by a relative increase in TG species with higher carbon numbers and greater degrees of unsaturation. Notably, TGs containing DHA consistently exhibited an upward trend, suggesting that DHA-enriched TG accumulation may contribute to TAC nephrotoxicity. These findings provide new insights into lipid metabolic remodeling that occurs in calcineurin inhibitor–associated renal injury. Detailed pathophysiology should be investigated by focusing on enzymatic and genetic factors. Future investigations using targeted lipidomics and human kidney samples with proteomic and transcriptomic approaches are warranted to validate these results and to explore their clinical implications.

## Data Availability

The datasets generated and/or analyzed during the present study are available from the corresponding author on request.

## Supplementary Materials

The following supporting information can be downloaded at: https://www.mdpi.com/article/10.3390/ijms26157549/s1.

## Abbreviations

AGC	automatic gain control
Cer	ceramide
CKD	chronic kidney disease
Cl	cardiolipin
DHA	docosahexaenoic acid
DG	diglyceride
DPA	docosapentaenoic acid
FFA	free fatty acid
HESI	heated electrospray ionization
LC	liquid chromatography
LPG	Lysophosphatidylglycerol,
MS	mass spectrometry
O-TG	ether-linked triacylglycerol
PC	phosphatidylcholine
PE	phosphatidylethanolamine
PG	Phosphatidylglycerol
PI	phosphatidylinositol
PS	phosphatidylserine
PUFA	polyunsaturated fatty acid
SM	sphingomyelin
TAC	tacrolimus
TG	triacylglycerol
