# Supplementary material for: DHA–Triacylglycerol Accumulation in Tacrolimus-Induced Nephrotoxicity Identified by Lipidomic Profiling
Source: Int J Mol Sci. 2025 Aug 5;26(15):7549. doi: 10.3390/ijms26157549 (PMC12347805; doi:10.3390/ijms26157549)
Supplement: Supplementary file 1 [file ijms-26-07549-s001.zip › ijms-3778426-supplementary.pdf]

Supplementary information for

DHA–Triacylglycerol Accumulation in Tacrolimus-Induced Nephrotoxicity Identified by Lipidomic Profiling

Sho Nishida [1,2], Tamaki Ishima [1], Daiki Iwami [2], Ryozo Nagai [3] and Kenichi Aizawa [1,4,*]

[1] Department of Translational Research, Clinical Research Center, Jichi Medical University Hospital, Shimotsuke 329-0498, Japan
[2] Division of Renal Surgery and Transplantation, Department of Urology, Jichi Medical University, Shimotsuke 329-0498, Japan
[3] Jichi Medical University, Shimotsuke 329-0498, Japan
[4] Clinical Pharmacology Center, Jichi Medical University Hospital, Shimotsuke 329-0498, Japan
* Correspondence: aizawa@jichi.ac.jp

Supplemental Table S1: Lipid classes detected under 10 metabolites

**Lipid class name**	**Number of metabolites**
Lysophosphatidylethanolamine	9
Cholesterol ester	8
Lactosyl ceramide	8
Globotriaosyl ceramide	8
Fatty acid	7
Glucosyl ceramide, Galactosyl ceramide	7
Coenzyme Q	4
Campesterol ester	3
Sulfatide	3
Acyl Carnitine	2
Bis(monooleoylglycero)phosphate	2
Lysophosphatidylinositol	2
Monoglyceride	1
Sitosterol ester	1
